# Study on risk factors for postoperative cough in children with hypertrophy of tonsils and adenoids

**DOI:** 10.3389/fped.2025.1730680

**Published:** 2026-01-13

**Authors:** Hong Mei Jiang, Xiao Yu Meng, Xing He Zhao, Zi Yu Shen, Xun Wu Dou

**Affiliations:** 1 Department of Pediatric Otorhinolaryngology, Suzhou Wujiang District Children’s Hospital, Suzhou, Jiangsu, China; 2 Department of Pediatric Otorhinolaryngology, Children’s Hospital of Soochow University, Suzhou, Jiangsu, China

**Keywords:** adenoids hypertrophy, pediatric sleep-disordered breathing, postoperative cough, risk factors, tonsil hypertrophy

## Abstract

**Background/aims:**

To investigate the risk factors for postoperative cough in children with tonsil and adenoid hypertrophy following tonsillectomy and adenoidectomy.

**Materials and methods:**

A total of 182 children with tonsil and adenoid hypertrophy treated in the Department of Otorhinolaryngology at the Wujiang District Children's Hospital of Suzhou City between January 2024 and December 2024 were selected. They were divided into groups based on the presence or absence of postoperative cough for a comparative study.

**Results:**

Statistically significant differences were found between the two groups regarding patient age, preoperative white blood cell levels, abnormal chest x-ray findings, and the presence of comorbid sinusitis or allergic rhinitis during the perioperative period. Multivariate logistic regression analysis identified younger age and comorbid sinusitis as independent risk factors.

**Conclusion:**

Younger age and comorbid sinusitis are significant risk factors influencing the occurrence of cough in children after tonsillectomy and adenoidectomy.

## Introduction

Sleep-disordered breathing (SDB) in children is classified based on the degree of upper airway narrowing and the limitation of gas exchange, representing a continuous disease spectrum. It progresses from initial primary snoring to upper airway resistance syndrome, and ultimately to obstructive sleep apnea (OSA). Relevant studies indicate that the prevalence of snoring in children is 8%, with approximately 1%–4% progressing to obstructive sleep apnea (1). This demonstrates that pediatric SDB is a significant cause of OSA in children. If left untreated, it can often lead to growth retardation, neurocognitive and behavioral abnormalities, and cardiovascular sequelae. Adenotonsillectomy (T&A) is the first-line treatment for SDB (2, 3).

Children with OSA are considered to be at higher risk for postoperative respiratory complications. Reports indicate that among children diagnosed with OSA undergoing adenotonsillectomy, 5.8%–26.8% experience postoperative respiratory complications, the most common being cough, compared to a rate of 1.3%–2.4% in the general pediatric population (4–8). A meta-analysis by Chen et al. (9) on tonsillectomy complications showed that children with OSA had nearly a five-fold greater probability of developing postoperative respiratory complications compared to children without OSA. Through a retrospective analysis of data from 182 children who underwent tonsil and adenoid removal, this study aims to investigate the factors associated with postoperative cough, thereby providing a reference for clinical perioperative management.

## Materials and methods

### General data

Data were collected from 182 children admitted to the Department of Otorhinolaryngology for tonsillar hypertrophy with adenoid hypertrophy who underwent tonsillectomy and adenoidectomy between January 2024 and December 2024. The cohort included 117 male children (64.28%) and 65 female children (35.72%). All data sources used in this study have been approved by the Ethics Committee of Wujiang District Children's Hospital in Suzhou City, and can be collected without patient informed consent (Ethics Code: 2025037).

#### Inclusion criteria

① Presence of clinical symptoms such as nocturnal snoring and/or mouth breathing, restless sleep or recurrent awakenings, and attention deficits; ② Physical examination confirming tonsillar hypertrophy accompanied by adenoid hypertrophy; ③ No significant improvement in symptoms after standard preoperative intervention therapy.

#### Exclusion criteria

① History of upper respiratory tract infection within 2 weeks prior to admission; ② Previous history of benign or malignant tumors of the tonsils or adenoids; ③ Presence of significant cough or sputum production before admission; ④ Severe systemic underlying diseases, such as severe cardiac, renal, or respiratory diseases; ⑤ Immune dysfunction or autoimmune diseases; ⑥ Contraindications to anesthesia; ⑦ Poor compliance or refusal to sign the informed consent form.

### Treatment method

With the child in the supine position and after stable general endotracheal anesthesia was achieved, routine disinfection, head draping, and toweling were performed. A Davis mouth gag was placed to expose the oropharynx. Using a low-temperature plasma radiofrequency ablation wand, an incision was made in the left anterior tonsillar pillar through the tonsil capsule. The tonsil was dissected along its capsule towards the posterior pillar until completely removed, with minor bleeding points coagulated using the plasma wand. The right tonsil was removed using the same method. A suction catheter was used to retract the soft palate via both nostrils. The adenoids were exposed using a 70° nasal endoscope, revealing hypertrophic adenoids obstructing over 50% of the posterior choanae, with partial extension into the choanae. The plasma wand was used to ablate the adenoid tissue, with the boundaries being the medial aspects of the torus tubarius, the inferior pole of the adenoids, and the roof of the posterior choanae. Care was taken intraoperatively to avoid injury to surrounding structures such as the dorsal surface of the soft palate, the pharyngeal orifice of the Eustachian tube, and the mucosa of the posterior choanae.

### Observation indicators

Postoperative cough occurrence (patients were assigned to the cough group if they experienced more than 5 episodes of coughing within 24 h after surgery; otherwise, they were assigned to the non-cough group) and the following parameters were recorded: patient gender, age, tonsil size grading, frequency of tonsillitis, presence of allergic rhinitis, presence of sinusitis, duration of illness, season of surgery, duration of surgery, intraoperative blood loss, bacterial culture results, preoperative complete blood count, and chest x-ray findings.

### Statistical methods

After initial data organization using Excel 2010, statistical analysis was performed with SPSS 27 software. Categorical data were analyzed using the Chi-square test. Normality testing was conducted for continuous data. Normally distributed data are presented as mean ± standard deviation, while non-normally distributed data are presented as median and interquartile range [*M* (P25–P75)]. Comparisons between two groups with normal distribution were performed using one-way ANOVA with *post-hoc* LSD test, and the Mann–Whitney *U* test was used for non-normally distributed data between two groups. A *P*-value <0.05 was considered statistically significant.

## Results

### Baseline characteristics and preoperative indicators

The patients were divided into two groups based on the presence or absence of postoperative cough: the cough group (*n* = 88) and the non-cough group (*n* = 94). A comparison of the baseline characteristics and preoperative examination results between the two groups revealed the following (details in Table 1): Statistically significant differences (*P* < 0.05) were observed between the two groups in terms of age, comorbid allergic rhinitis, comorbid sinusitis, preoperative white blood cell levels, and abnormal chest x-ray findings.

**Table 1:** Baseline characteristics and preoperative profiles of the two groups.

Variables	Cough group (*n* = 88)	Non-cough group (*n* = 94)	*Z*/*x*^2^	*P*
Age (years)♦	6.375 (4.5, 8.06)	7.42 (5.29, 9.60)	−2.672	0.006
Gender (Male:Female)	60:28	57:37	1.126	0.353
Disease duration (years)	6 (3, 12)	6 (3, 6)	1.19	0.373
Comorbid allergic rhinitis, n♦	46	69	−2.946	0.03
Comorbid sinusitis, n♦	38	16	3.85	<0.001
Preoperative white blood cell count (×10⁹/L)♦	7.31 ± 2.00	6.65 ± 1.62	−2.419	0.017
Preoperative eosinophil count (×10⁹/L)	0.18 ± 0.24	0.24 ± 0.68	1.25	0.213
Preoperative abnormal chest x-ray findings, n♦	4	1	4.21	<0.001

Items with “♦” refer to *P* < 0.05, which denotes statistical significance.

Analysis of the medical history data of children who underwent adenotonsillectomy yielded the following results (details in Table 2): Preoperative tonsil size grading showed no statistically significant impact on the occurrence of postoperative cough. In contrast, preoperative bacterial culture results and the frequency of inflammatory episodes were statistically significant factors (*P* < 0.05).

**Table 2:** Analysis of preoperative factors associated with postoperative cough in children after adenotonsillectomy.

Factor category	Number of cases (*n* = 88)	Percentage (%)	*Z*/*x*^2^	*P*
Tonsil size grading				
I	6	6.8%	−0.767	0.104
II	54	62.4%		
III	28	31.8%		
Frequency of inflammatory episodes (/year) ♦				
0–3	49	55.7%	11.3	<0.001
4–6	21	23.9%		
7–10	18	20.4%		
Bacterial culture ♦			80.182	<0.001
Positive	2	2.3%		
Negative	86	97.7%		

Items with “♦” refer to *P* < 0.05, which denotes statistical significance.

### Analysis of intraoperative factors

**Figure 1:**
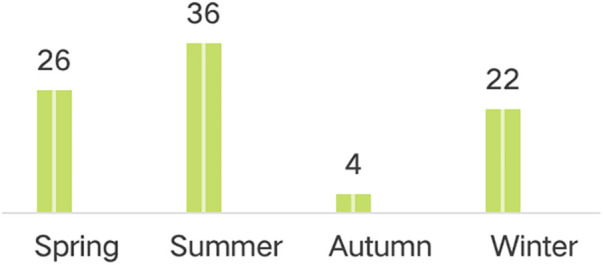
Distribution of surgical seasons in the cough group.

A comparison of intraoperative conditions between the cough group and the non-cough group was performed, focusing on the factors of operative duration and intraoperative blood loss. Additionally, a separate analysis of the season of surgery was conducted for the cough group. The results are as follows (details in Table 3 and Figure 1): No significant differences were observed between the two groups regarding operative duration or intraoperative blood loss, with these findings lacking statistical significance. However, the distribution of the season of surgery within the cough group was as follows: 26 cases in spring, 36 in summer, 4 in autumn, and 22 in winter. Chi-square analysis yielded a value of *X*^2^ = 24.364 with *P* < 0.01, indicating a statistically significant difference.

**Table 3:** Comparison of intraoperative findings between the two groups.

Variables	Cough group (*n* = 88)	Non-cough group (*n* = 94)	*Z*/*x*^2^	*P*
Operative duration (minutes)	27.31 ± 7.89	25.31 ± 6.19	1.12	>0.05
Intraoperative blood loss (mL)	5.22 ± 1.39	5.43 ± 3.17	1.01	>0.05

### Results of multivariate logistic regression analysis

A multivariate logistic regression analysis was performed, incorporating the factors of age, frequency of inflammatory episodes, comorbid sinusitis, comorbid allergic rhinitis, and white blood cell level. The results are presented in Table 4. The regression equation derived from the logistic regression analysis was Logit(*P*) = −0.53 − 0.123*X*_1_ + 0.041*X*_2_ + 1.020*X*_3_ + 0.142*X*_4_ − 0.113*X*_5_ − 0.53*X*_6_. The analysis revealed that younger age and comorbid sinusitis were significant risk factors for cough following adenotonsillectomy, with odds ratios (OR) of 0.884 and 2.774, respectively (*P* < 0.05).

**Table 4:** Multivariate logistic regression analysis of postoperative cough following adenotonsillectomy.

Variables	Regression coefficient (*B*)	S.E.	Wals	df	Sig.*P*	OR	CI for EXP(*B*) lower 95%	CI for EXP(*B*) upper
Age	−0.123	0.065	3.535	1	0.047	0.884	0.778	1.005
Inflammatory episodes	0.041	0.052	0.634	1	0.426	1.042	0.942	1.153
Comorbid sinusitis	1.020	0.509	4.024	1	0.045	2.774	1.024	7.516
White blood cell count	0.142	0.093	2.337	1	0.126	1.152	0.961	1.382
Comorbid allergic rhinitis	−0.113	0.472	0.057	1	0.811	0.893	0.354	2.252
Constant	−0.53	0.960	0.304	1	0.581	0.589		

## Discussion

Clinical observation indicates that cough is a highly prevalent complication following pediatric adenotonsillectomy; however, its associated risk factors and clinical implications have not been systematically investigated. Unlike postoperative hemorrhage, which has garnered significant attention due to its severity, the impact of postoperative cough on recovery quality has not been adequately addressed. This study aimed to address this gap in knowledge. Postoperative cough may elevate the risk of postoperative hemorrhage, as suggested by our data where the incidence was 3/88 in the cough group compared to 1/94 in the non-cough group. Furthermore, it negatively impacts postoperative pain, recovery, hospital stay, and the emotional and psychological state of both the child and parents, necessitating adequate experience from clinicians and nursing staff for appropriate management. Most cough episodes occur within the first 24 h post-surgery, lasting an average of 5–7 days, and typically resolve with nebulization therapy and symptomatic antitussive treatment.

To explore potential factors associated with cough in children undergoing plasma-mediated adenotonsillectomy, this study investigated observation indicators including gender, age, tonsil size grading, frequency of tonsillitis, presence of allergic rhinitis, presence of sinusitis, disease duration, surgical season, operative duration, intraoperative blood loss, bacterial culture results, preoperative complete blood count, and chest x-ray findings.

**Figure 2:**
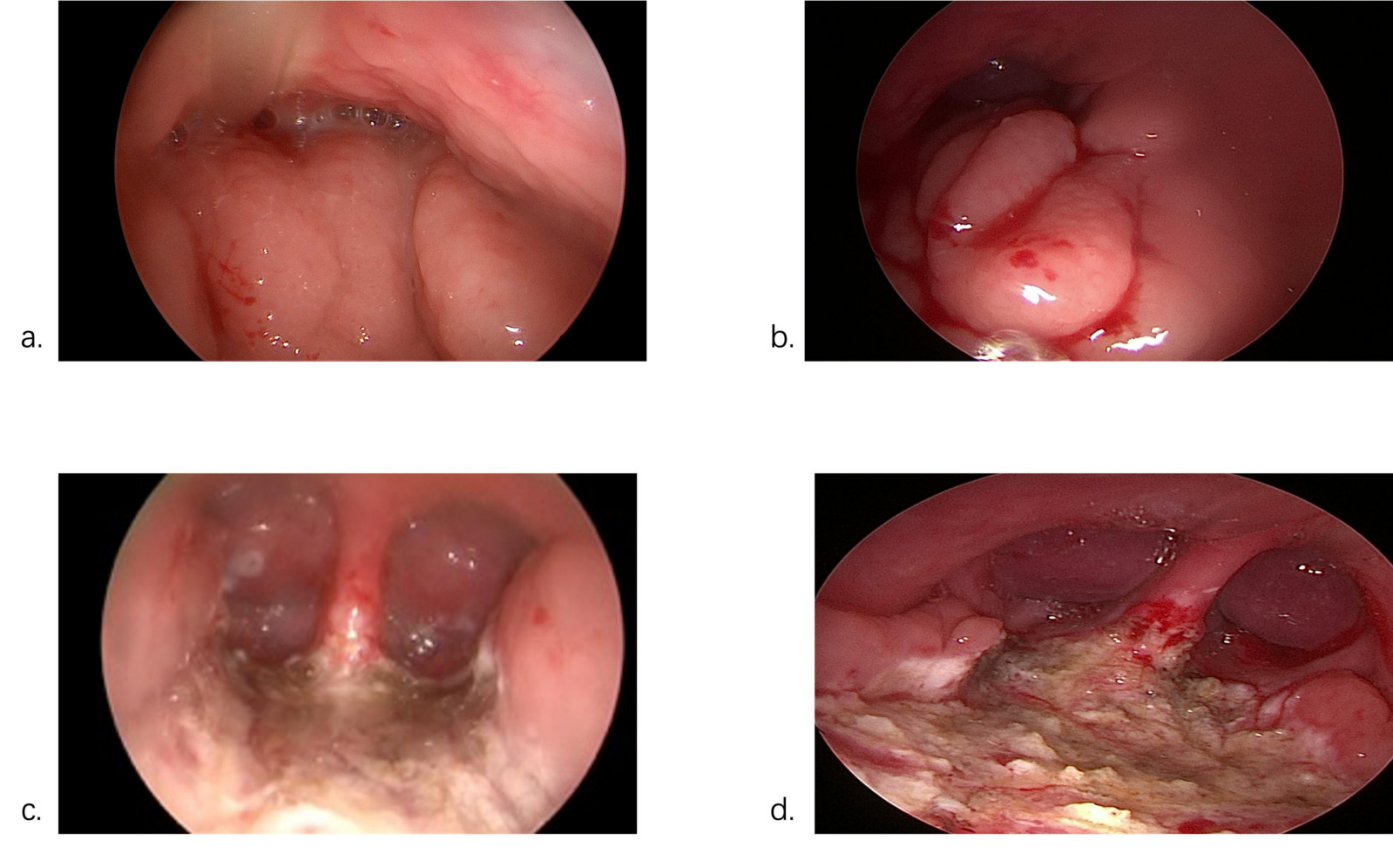
Intraoperative comparison of children with comorbid sinusitis. **(a,c)** Preoperative and postoperative endoscopic findings in children with comorbid sinusitis; **(b,d)** Preoperative and postoperative endoscopic findings in children without comorbid sinusitis).

Analysis of the baseline characteristics and preoperative examination results indicated a correlation between patient age and the occurrence of postoperative cough, with younger children exhibiting a higher likelihood of developing cough. Furthermore, the presence of comorbid allergic rhinitis and sinusitis also demonstrated statistical significance. Sinusitis was diagnosed according to the criteria outlined in the “2025 Italian Intersociety Consensus on the Treatment of Pediatric Sinusitis” (10). In this study, direct intraoperative comparison of patients with comorbid sinusitis (Figure 2) revealed nasal mucosal edema, congested and hypertrophied inferior turbinates, mucopurulent discharge in the nasal cavity and middle meatus, adenoid hypertrophy obstructing >50% of the posterior choanae, and abundant mucopurulent secretions adherent within the residual space (Figure 2a). Postoperatively, these children exhibited retained mucopurulent secretions in the various nasal meati (Figure 2c). Pre-existing sinusitis leads to the accumulation of nasal and sinus secretions. This facilitates the dissolution of harmful airborne chemicals and the proliferation of pathogenic microorganisms within the secretions, creating reservoirs for bacterial colonization. This process irritates the sinus ostium mucosa, causing edema, ostial obstruction, and impaired sinus drainage. Consequently, the respiratory tract initiates protective mechanisms, resulting in cough, which aligns with the findings of this study. Although the preoperative white blood cell counts in both groups were within normal limits, levels were higher in the cough group. Whether this finding is directly related to comorbid sinusitis requires further investigation.

Allergic rhinitis (AR) is a major inflammatory disease of the respiratory tract in children (11). Its prevalence is increasing annually, with the prevalence rate among children in China being approximately 18.46%, albeit with significant regional variations (12). OSA has a high comorbidity rate with conditions such as AR and asthma, and the pathogenesis and progression of these diseases interact. AR not only causes nasal congestion but may also influence the onset and progression of OSA by affecting airway resistance due to chronic inflammatory stimulation. AR and OSA frequently coexist and are closely interrelated. The incidence of AR in children with OSA is 2.12 times higher than in the general population. AR imposes a substantial disease burden. Studies have found that children with AR often have comorbid conditions such as adenoid hypertrophy, chronic sinusitis, otitis media, and OSA. Improvement of these related comorbidities can contribute to better control of AR symptoms; therefore, introducing reasonable and effective treatment strategies is crucial for enhancing AR management. This study indicated that the presence of AR symptoms in children had a certain significance regarding postoperative cough. However, eosinophil levels, a marker associated with allergy, did not show statistical significance, necessitating further studies with larger sample sizes and additional indicators such as IgE, mast cells, and interleukins.

Beyond the factors discussed above, the preoperative frequency of inflammatory episodes, bacterial culture results, and the season of surgery also proved significant. In this study, bacterial cultures were primarily obtained from throat swabs and blood cultures. Two cases were positive, identifying Haemophilus parainfluenzae. Haemophilus parainfluenzae is a Gram-negative coccobacillus belonging to the genus Haemophilus. It commonly colonizes the human respiratory mucosa and can cause infections under certain conditions, frequently associated with respiratory tract infections (such as otitis media, sinusitis, pneumonia) and potentially other infections (such as septicemia, meningitis), especially in immunocompromised individuals. It is closely related to sinusitis, as discussed previously. However, due to the inherent time delay associated with bacterial culture, it lacks clinical utility for guiding preoperative prophylaxis and was therefore not included in the multivariate analysis. The season of surgery was defined as follows: Spring (March, April, May), Summer (June, July, August), Autumn (September, October, November), and Winter (December, January, February). The results showed a significantly higher number of surgeries performed in summer compared to other seasons, primarily attributable to the “seasonality” of pediatric surgical procedures in the studied region, where the summer vacation period is substantially longer than the winter break, leading to a higher volume of pediatric consultations and surgeries during that time. Consequently, this factor was also excluded from the multivariate analysis.

Based on the multivariate logistic regression analysis, younger age and comorbid sinusitis were identified as significant risk factors for cough following adenotonsillectomy. Therefore, implementing preventive measures against cough at an early stage is particularly important for younger children with comorbid sinusitis. Currently, the primary clinical approach involves initiating nebulization therapy early in the postoperative period to prevent the onset of cough and its potential to trigger other serious complications.

## Data Availability

The original contributions presented in the study are included in the article/Supplementary Material, further inquiries can be directed to the corresponding author.
